# The Oleofobization of Paper via Plasma Treatment

**DOI:** 10.3390/polym13132148

**Published:** 2021-06-29

**Authors:** Matic Resnik, Eva Levičnik, Žiga Gosar, Rok Zaplotnik, Janez Kovač, Jernej Ekar, Miran Mozetič, Ita Junkar

**Affiliations:** 1 Department of Surface Engineering, Jožef Stefan Institute, Jamova 39, 1000 Ljubljana, Slovenia; eva.levicnik@ijs.si (E.L.); rok.zaplotnik@ijs.si (R.Z.); janez.kovac@ijs.si (J.K.); jernej.ekar@ijs.si (J.E.); miran.mozetic@ijs.si (M.M.); ita.junkar@ijs.si (I.J.); 2 Elvez d.o.o., Ulica Antona Tomšiča 35, 1294 Višnja Gora, Slovenia; ziga.gosar@elvez.si

**Keywords:** oleofobization, paper, cellulose, plasma, HMDSO

## Abstract

Cellulose is a promising biomass material suitable for high volume applications. Its potential lies in sustainability, which is becoming one of the leading trends in industry. However, there are certain drawbacks of cellulose materials which limit their use, especially their high wettability and low barrier properties, which can be overcome by applying thin coatings. Plasma technologies present a high potential for deposition of thin environmentally friendly and recyclable coatings. In this paper, two different plasma reactors were used for coating two types of cellulose-based substrates with hexamethyldisiloxane (HMDSO). The changes in surface characteristics were measured by atomic force microscopy (AFM), scanning electron microscopy (SEM), surface free energy and contact angles measurements, X-ray photoelectron spectroscopy (XPS), and secondary ion mass spectrometry (SIMS). Successful oleofobization was observed for an industrial scale reactor where pure HMDSO was used in the absence of oxygen.

## 1. Introduction

High-volume industries are seeking new alternative materials to become sustainable and decrease pollution. One of the most promising materials being implemented is cellulose, a biomass derived raw material which is abundant, renewable, inexpensive, and biodegradable. Thus, the use of cellulose is expected to increase [1], as it has high potential of application in various industries. Major industries implementing materials made from cellulose nanofibrils (CNF) and microfibrillated cellulose (MFC) are the wood, paper, and textile industries. However, only cellulose with appropriately tailored surface properties can be used for separating oil from water [2], food packaging [3], self-cleaning surfaces [4,5], microchips [6], antibacterial agents [7], etc. Various surface finishing procedures were proposed and studied to achieve hydrophobic or oleophobic surface properties for cellulose-based products. They can be divided into wet chemical methods (like liquid spray-coating [8,9], dip-coating [10], sol-gel [11], etc.), where usually organic solvents are required [12]; or dry methods, that are more environmentally friendly, such as liquid flame spray [13] or plasma-based techniques [14,15].

PECVD (plasma enhanced chemical vapor deposition) is a commonly used technique for depositing thin layers [16] on surfaces. Various precursors can be used to apply Si-containing thin films, with hexamethyldisiloxane (HMDSO) being among the most popular ones. HMDSO is a non-toxic liquid [17] and an easy-to-handle monomer; however, deposition of HMDSO films by plasma polymerization is hard to control, mainly due to the diversity of functional groups produced by the multitude of possible chemical reactions [18]. The structure and composition of plasma polymerized HMDSO films have been widely studied for its application in biocompatible coatings [19,20,21], barrier and protective coatings [20,22], as well as for water repellence [23,24]. It is used to deposit thin films ranging from HMDSO-like polymer films to almost pure SiOx films. The HMDSO plasma polymerization of cellulose-based products, such as paper for food packaging, has considerable benefits compared to other coating methods. The quantity of deposited material by PECVD is orders of magnitude smaller, making the process more cost-efficient and the product recyclable [3]. With the recent advantages in plasma techniques, both low-pressure [4,7] and atmospheric pressure [2,3,25] plasma can be successfully employed. The initial investment in PECVD systems in case of low-pressure plasma might be high, while the operating costs are low, and any high-volume industry should on the long run profit using these systems.

HMDSO is a liquid at atmospheric pressure. It has a high vapor pressure (about 50 mbar at room temperature) and can, therefore, be introduced into the processing chamber via a leak valve or gas flow regulator. In the processing chamber, a non-equilibrium gas plasma is created by discharge, where free electrons (with temperature of approximately 10,000 K) cause radicalization and ionization of the precursor. Plasma is often excited by high-frequency discharges [26]. The reactive particles disperse inside the chamber and eventually reach a surface where they adhere with a certain probability. Substrates are often activated before application to improve adhesion. Different coatings can be applied depending on the plasma parameters.

One extreme is a coating of polydimethylsiloxane like films. Such a coating is obtained at a very low power density (in order to preserve the original composition of HMDSO) and in the absence of other gases. The other extreme is a thin layer of silicon dioxide that grows when oxygen-containing gas is present in the chamber, like water vapor, which is usually present in vacuum chambers, or sometimes oxygen is added intentionally to form purer SiOx [27,28]. Between these extremes, all types of coatings can be achieved, depending on the processing parameters. The flow of radicals to the surface (and thus the rate of deposition) obviously increases with increasing pressure and power density. At elevated pressure (more than 10 Pa), however, the radicals begin to agglomerate already in the gas phase, so that the coating becomes granular, which is often considered harmful in industrial systems. If the power density is increased, the dissociation of the precursor is intense, so that carbon atoms or even dimers can be incorporated into the SiOx film, making it less transparent. Another obstacle is the uniformity of the plasma; dissociation and ionization events are more intense closer to the electrodes, so the SiOx film is applied mainly to the electrodes instead of the substrates [29].

The work presented herein aims to apply Si-coatings using HMDSO deposited by plasma to prepare surfaces suitable for food-packaging applications. Two different low-pressure plasma systems were used, the small-scale laboratory reactor and large-scale industrial reactor, to study the effects of plasma treatment on two different types of papers. The influence on surface chemistry, morphology, and hydrophilic and oleophobic properties were studied and assessed for its industrial applicability. The main goal was to improve the oleophobicity of the paper substrates by Si-based coatings. Such modification might be of great interest to high-volume industries, such as the food packaging industry. Cellulose has all of the benefits sought by such an industry, except for the oleophobicity and desired barrier properties.

## 2. Materials and Methods

### 2.1. Paper Samples

Two different types of pre-coated papers were used for surface modification with HMDSO. The first paper was made from a combination of softwood (eucalyptus) and hardwood with production rests, and pre-coated with CaCO_3_, kaolin fillers, and a latex binder. For the purpose of this text, this type of paper will be referred to paper 1. The other paper, referred as paper 2, was made from deinked pulp, mechanical pulp, and consisted of 18–24% inorganic parts, and pre-coated with starch. This type of paper had a much higher organic part in the coating compared to paper 1. Both papers were used for the deposition of HMDSO coating on the surface to study the coating morphology, chemical composition, and wettability, as well as oleophobic properties of the coating. The papers were treated in A4 format and analyzed with different surface analyzing techniques, as described in the following sections.

### 2.2. Plasma Enhanced Chemical Vapor Deposition (PECVD)

Two different types of plasma reactors were used for modification of papers: the laboratory reactor operating at microwave (MW) discharge, and the industrial reactor operating at radiofrequency (RF) discharge. Due to the specifics of the two plasma systems, different treatment conditions were used for surface modification and presented in more detail below.

#### 2.2.1. Laboratory Reactor

A low-pressure microwave (MW) plasma was generated in the LA400 plasma system (SurfaceTreat, Turnov, Czech Republic) [30]. A double stage rotary vacuum pump with 65 m^3^/h nominal pumping speed was used to evacuate the 64 L processing chamber to a base pressure of 1 Pa. The processing chamber made of aluminum had a magnetron mounted on top, with microwaves entering the chamber through a quartz glass. The heated HMDSO container was mounted close to the chamber and leaked into it. The flow of HMDSO and oxygen, the MW generator’s power, the distance between the magnetron and the sample, and the exposure time were varied to reach the optimal parameters.

The samples of paper 1 and 2 were prepared by 10 min plasma deposition with MW plasma, where the feeding gas was a mixture of HMDSO and O_2_ (ratio 7:1, respectively) at 50 Pa of combined pressure and MW power of 200 W. The distance between the magnetron and the sample was approximately 0.2 m.

#### 2.2.2. Industrial Reactor

An industrial scale reactor for PECVD was used for depositing HMDSO on paper samples. The reactor was cylindrically shaped with a 0.95 m radius and a height of 1.8 m. Multiple vacuum pumps were used for sustaining low pressures inside the reactor. Diffusion pumps were supported by roots and rotary pumps and a cold trap, together reaching a base pressure in the range of 0.01 Pa. A perforated tube was used to evenly distribute the gas fed into the reactor via flow controllers. Plasma at low pressure was sustained by an asymmetric capacitively coupled RF discharge. Two powered electrodes (0.5 m^2^ each) were located close to the grounded reactor housing (approximately 17 m^2^). Powered electrodes were connected to a RF generator with adjustable power output (up to 8 kW) operating at the frequency of 40 kHz. More about this reactor, including its schematic, can be found in an earlier paper by Gosar et al. [22,31].

The samples of paper 1 and 2 were mounted onto planetary stands with two rotational movements, one around its own axis and another one around the center of reactor. This kind of movement should ensure equal treatment for all samples. Afterwards, the reactor was pumped to a base pressure, and a 20-min cycle of PECVD of HMDSO began.

### 2.3. Surface Morphology Analysis

#### 2.3.1. Scanning Electron Microscopy

Morphological properties of the samples were analyzed using scanning electron microscopy (SEM). Approximately 5 × 5 mm^2^ pieces of treated and untreated paper were cut from the material. They were attached onto aluminum stubs using conductive carbon tape, and their edges connected to the stub surface using carbon paste and coated with a thin gold layer (10–12 nm thick) using a Balzers SCD 050 sputter coater (BAL-TEC GmbH, Schalksmühle, Germany). The SEM images were obtained using a Jeol JSM-7600F Schottky Field Emission scanning electron microscope (Jeol Ltd., Tokyo, Japan).

#### 2.3.2. Atomic Force Microscopy

Changes in surface morphology of paper samples were analyzed by Atomic force microscope (AFM) Solver PRO (NT-MDT, Moscow, Russia) in non-contact mode in air. Samples were cut into small pieces, and the surface was scanned by a standard Si cantilever with a force constant of 22 Nm^−1^ and at a resonance frequency of 325 kHz. The cantilever’s tip radius was 10 nm, the tip length was 95 µm, and the scan rate was set at 1.2 Hz. Every measurement was repeated at least five times. Average surface roughness (Sa) was measured from representative images on 5 × 5, 2 × 2, and 1 × 1 µm^2^ areas with the Nova AFM software (NT-MDT, Moscow, Russia). Paper 2 in untreated state was too rough to be analyzed with our AFM device.

### 2.4. Surface Free Energy and Contact Angle

The surface wettability was performed with the Drop Shape Analyser DSA-100 (Krüss GmbH, Hannover, Germany) by a sessile drop method to measure a static contact angle. Surface wettability was analyzed immediately after plasma treatment. The relative humidity was around 45% and the operating temperature was 21 °C, which did not vary significantly during continuous measurements.

**Figure 1:**
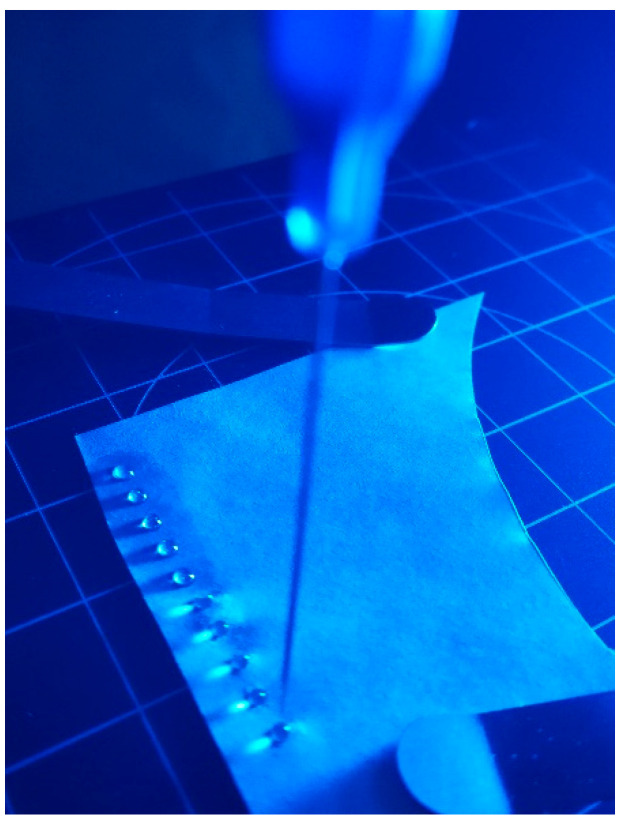
An array of distilled water and diiodomethane drops with a volume of 1.5 µL applied to the paper surface with a distance of 3 mm between individual drops.

The Krüss GmbH device for measuring surface wettability had a platform, which automatically moved by the X and Y axes (Figure 1). After setting X and Y positions for the simultaneous deposition of a drop, a contact angle was recorded. Surface energy was determined from contact angle measurements. According to the Tappi T 5580m 97 standard, the OWRK (Owens, Wendt, Rabel and Kaelble) fitting method with water (2.5 µL drop of deionized water) and diiodomethane (1.5 µL drop of diiodomethane) was used by the Drop Shape Analyser. Additionally, pumpkin oil (1.5 µL drop manually added by a syringe) was used to determine oleophobic properties of the coating.

### 2.5. Surface Chemistry Analysis

#### 2.5.1. X-ray Photoelectron Spectroscopy

The X-ray photoelectron spectroscopy (XPS or ESCA) analyses of papers were carried out by a PHI-TFA XPS spectrometer produced by Physical Electronics Inc. (Physical Electronics Inc., Chanhassen, MN, USA). Samples were placed on metallic support and introduced in ultra-high vacuum spectrometer. The analyzed area was 0.4 mm in diameter, and the analyzed depth was about 3–5 nm. This high surface sensitivity is a general characteristic of the XPS method. Sample surfaces were excited by X-ray radiation from monochromatic Al source at a photon energy of 1486.6 eV. The high-energy resolution spectra were acquired with an energy analyzer operating at a resolution of about 0.6 eV and pass energy of 29 eV. During data processing, the spectra from the surface were aligned by setting the C 1s peak at 285.0 eV, characteristic for C–C bonds. The accuracy of binding energies was about ±0.3 eV. Quantification of surface composition was performed from XPS peak intensities considering relative sensitivity factors provided by the instrument manufacturer [32]. Three different XPS measurements were performed on each sample and average composition was calculated.

In order to analyze thickness and in-depth distribution of elements in the SiO_2_ films, the XPS depth profiling was performed in combination with ion sputtering. Ar ions of 4 keV energy were used. The velocity of the ion sputtering was estimated to be 2.0 nm/min, calibrated on the SiO_2_ structure of the known thickness.

#### 2.5.2. Secondary Ion Mass Spectrometry

TOF-SIMS (Time-of-flight secondary ion mass spectrometry) analyses were made on the TOF.SIMS 5 instrument produced by the IONTOF company (IONTOF GmbH, Münster, Germany). As the analytical beam, we used Bi^3+^ primary ions with energy of 30 keV. Analytical depth with the settings used was around 2 nm and detection limits were around 1 ppm of species of interest in the sample. As the paper samples were nonconductive, the low energy electron beam had to be applied to neutralize excessive positive charge.

Positive and negative surface spectra were recorded in the areas of 250 × 250 µm^2^ while measuring secondary ions in the *m/z* range from 0 to 875. Mass resolution (m/Δm) was between 3500 and 7000, depending on the peak of interest. Micrographs of positive secondary ions of interest were also recorded in the areas of 500 × 500 µm^2^. The lateral resolution of micrographs was 180 nm and quality was 512 × 512 pixels. Depth profiles of positive secondary ions were recorded on the 100 × 100 µm^2^ areas while rastering the 1 keV O^2+^ primary ion sputter beam over the area of 400 × 400 µm^2^.

## 3. Results

### 3.1. Surface Morphology

**Figure 2:**
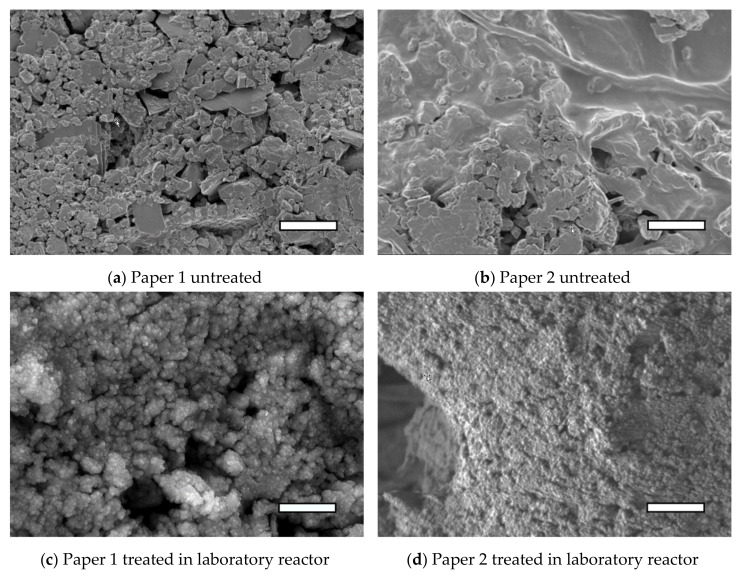

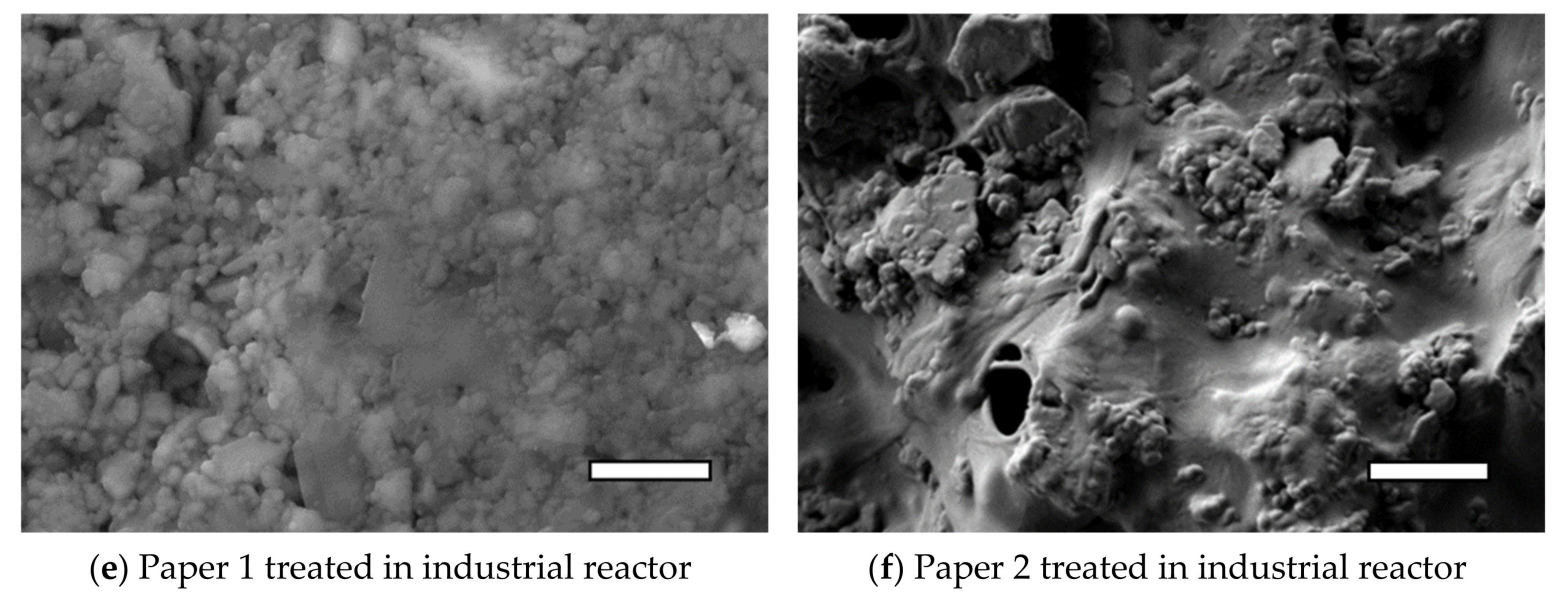
Comparison of SEM, higher magnification coatings on paper 1 and 2 with two different plasma systems. On the left there is paper 1, (**a**) untreated, (**c**) treated in laboratory reactor, (**e**) treated in industrial reactor. On the right there is paper 2, (**b**) untreated, (**d**) treated in laboratory reactor, (**f**) treated in industrial reactor. White bar represents 1 µm scale.

Untreated paper 1 and paper 2 have different surface morphologies, as presented in Figure 2. On paper 1, a grain like structure is observed, with visible kaolin particles; while on paper 2, the surface exhibits grain like structure together with smoother parts that can be ascribed to the organic parts of the coating. Figure 2a represents a sample of paper 1 before plasma treatment. Microparticles with relatively wide size distribution were observed. Larger kaolin particles were also clearly visible, with their distinctive edges and flat surfaces. There were many empty spaces between the grains in the case of paper 1, which makes it porous. In case of paper 2 (Figure 2b), microparticles were not so well observed, as it seemed they were covered with the organic parts of the coating. However, in this case, the microroughness, according to AFM, was much higher as it was not possible to obtain useful AFM data on these surfaces.

Interestingly, after plasma treatment, similarities can be observed for both paper samples. After the deposition of HMDSO in a laboratory plasma system, the entire surface was covered in what appeared to be fine nano grains (Figure 2c,d). Many of the former grain boundaries between macroparticles and empty spaces seemed to be covered with a HMDSO coating. In the case of plasma treatment of paper 1 in the laboratory reactor (Figure 2c), the coating was not completely flat and dark pores were still present, probably due to a relatively thin layer of HMDSO coating (a few nanometers). Paper 2 treated in the laboratory reactor is shown in Figure 2d, where a unified fine-grained surface was clearly visible. The HMDSO coating seemed to fill even the small gaps between the pores.

Further on, the SEM images of both samples of paper treated in the industrial reactor reveal what appeared to be a thicker and more dense coating compared to the one from the laboratory reactor. The surface of paper 1 treated in the industrial reactor exhibited a densely packed grain-like structure, which seemed to have fewer gaps in the structure (Figure 2e) compared to surfaces treated in laboratory plasma reactor. Similarly, a dense coating with less pronounced grain-like structure was observed on paper 2 treated in the industrial reactor (Figure 2f). The sample of paper 2 treated in the industrial reactor for 20 min appears to be fully covered with a HMDSO like coating, and only random clusters of grains can be observed under the HMDSO coating.

Similar results were reported by Babaei et al. [3], where the samples of Kraft paper were exposed to atmospheric pressure plasma polymerization of HMDSO under helium (He). Their sample had microfibril structures visible at higher magnifications, which also remained visible after being uniformly covered with HMDSO grains in the process of PECVD. According to the results of this study, the coating was comprised of SiOCH, which was not uniformly distributed. The amount of organosilicon coating gradually decreased in the direction away from the HMDSO gas inlet. Hydrophobic surfaces were obtained, with a water contact angle of about 140°.

**Figure 3:**
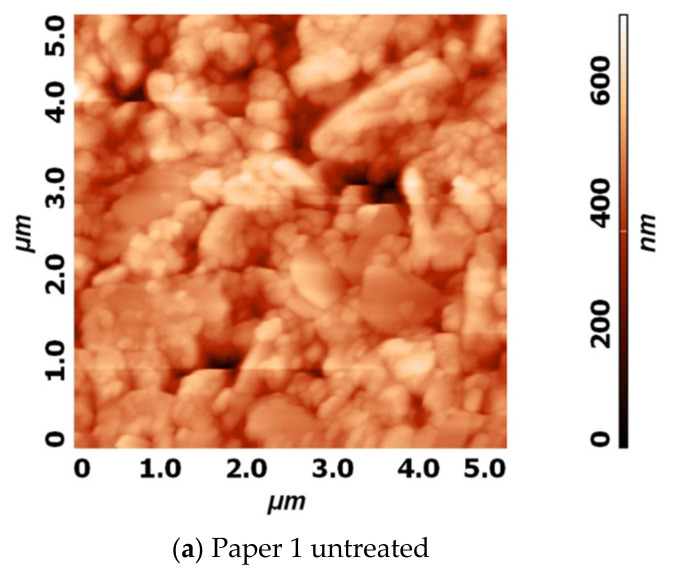

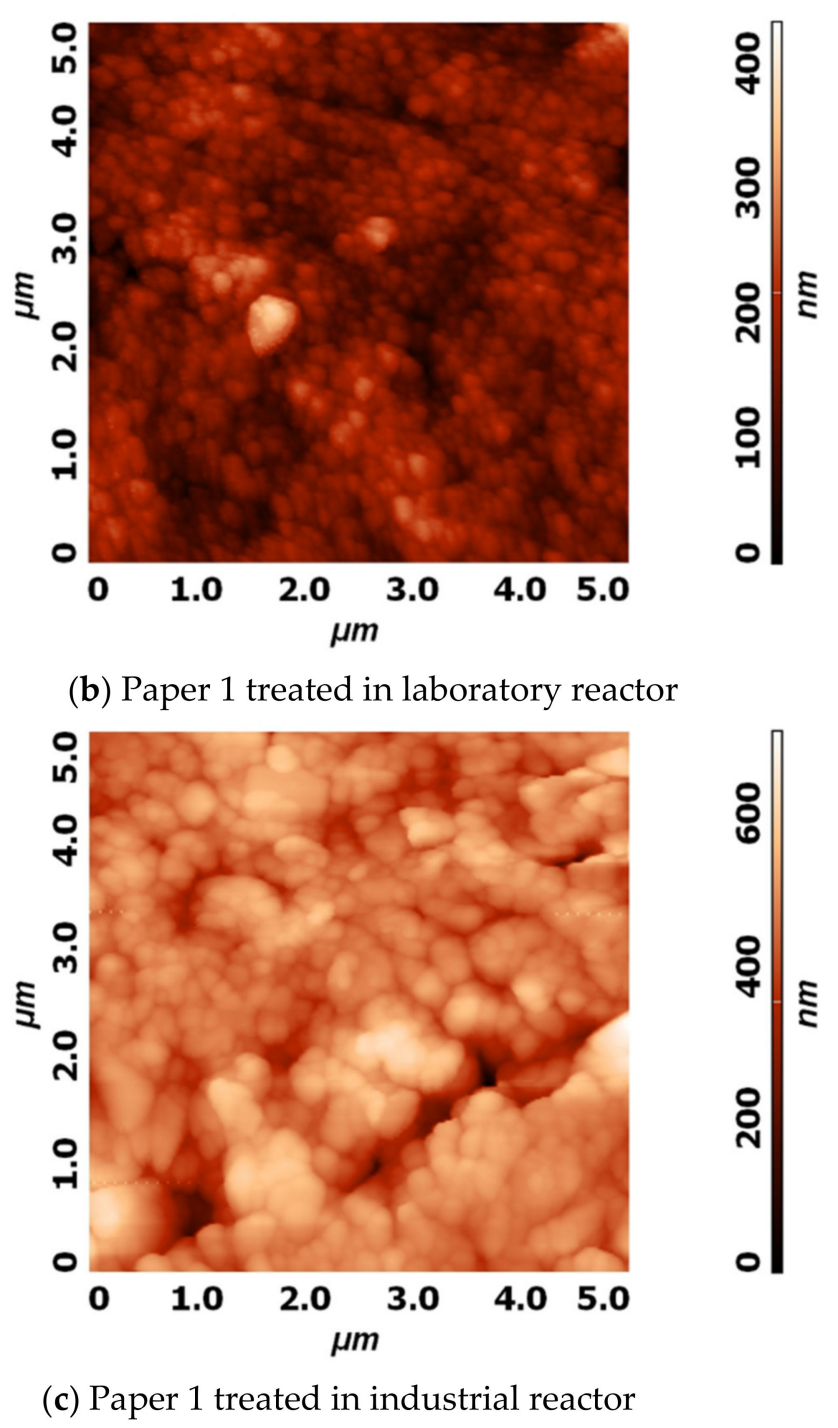
AFM images of height profiles on paper 1 before (**a**) and after plasma treatment in laboratory (**b**) and industrial (**c**) reactor.

The AFM images conducted on paper 1 provided more detailed analysis of surface morphology. After plasma treatment in both plasma systems, fine grain structures were observed on the paper surface, which seemed to cover the initial paper micro topography uniformly. Untreated paper 1 had a relatively wide distribution of micro and nano particles, with gaps between the grain boundaries (Figure 3a). In contrast, paper 1 treated in laboratory reactor was covered with smaller and more defined grains, with uniform grain size distribution ranging from 100 to 200 nm. The grain borders were visible and seemed to also cover the spaces between the micro particles. The grain-like structure that was observed in the case of paper 1 treated in industrial reactor was denser, with bigger grains ranging from 200 to 600 nm. The average roughness was also analyzed. However, due to high nonuniformity of the surface, especially due to its microstructure, it was hard to compare changes in roughness between the samples. In a study by Nättinen et al., where HMDSO was deposited onto LDPE (low density polyethylene) and cotton fabric for its hydrophobic effect [33], the shape and size of grains reported were similar to the ones presented herein after laboratory-plasma treatment.

### 3.2. Surface Free Energy and Hydrophilic/Oleophobic Properties

According to the wettability analysis, two different trends were observed after deposition of the HMDSO coating. According to the wettability data, presented in Table 1, the untreated paper 1 and 2 are hydrophobic. After plasma treatment in the laboratory reactor, where oxygen was also present, both papers became hydrophilic and the surface free energy rose. However, this was not the case with the industrial scale reactor, where only HMDSO was present during plasma treatment (no addition of oxygen). After industrial plasma treatment, paper 1 became significantly more hydrophobic, while paper 2 kept its hydrophobicity. It should be emphasized that nanotopographic features (according to SEM) on both papers were similar, while the initial microtopography was different, which could partially influence the wettability. Wettability changes were also observed for Si–O–Si, Al–O, and Zr–O ceramic-based sol-gel treated CNF films by Vartiainen et al., who reported an increase in water contact angle from 54 to 102 degrees after the coating [34]. In this study, decreased water vapor transmission was also reported. Compared to the untreated sample, the surface free energy dropped close to 5 and 2-fold for industrially treated paper 1 and paper 2, respectively. Similar behavior was also observed by Vartiainen et al., where roll-to-roll atmospheric plasma deposition of HMDSO onto cellulose films was studied. Vartiainen et al. reported water contact angles of 23° and 103° for untreated cellulose nanofibrils and coated cellulose nanofibrils, respectively [1].

**Figure 4:**
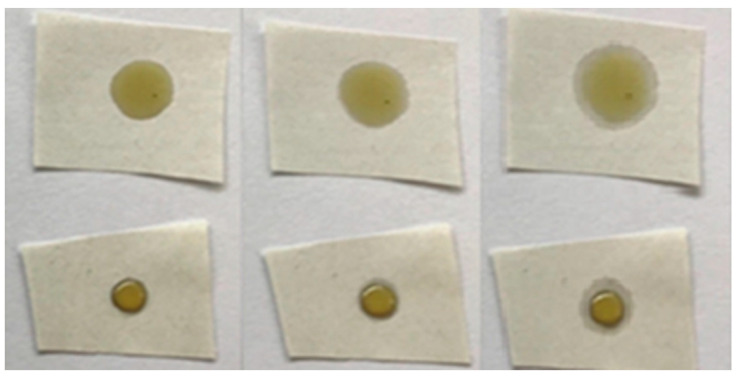
Time lapse of untreated (**above**) and industrial-plasma treated (**below**) paper 1, from left to right, immediately after plasma treatment, 10 min after plasma treatment, and 24 h after plasma treatment.

**Figure 5:**
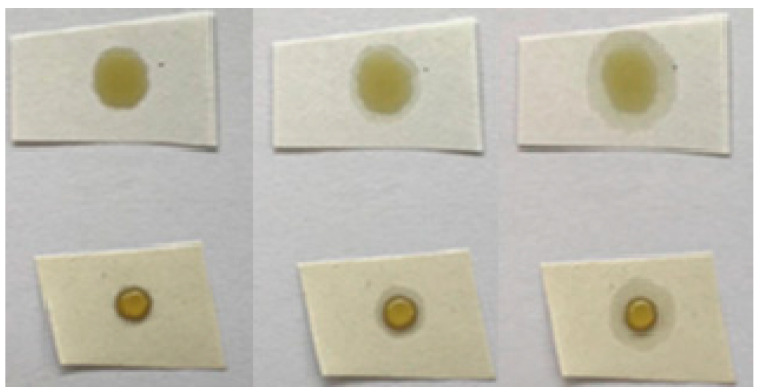
Time lapse of untreated (**above**) and industrial-plasma treated (**below**) paper 2, from left to right, immediately after plasma treatment, 10 min after plasma treatment, and 24 h after plasma treatment.

The increase in oleophobic properties was not observed for untreated and laboratory-plasma treated samples, as the oil was fully spread on the paper. In the case of industrial-plasma treatment, the HMDSO coating on both papers seemed to prevent the oil from penetrating into the paper, as shown in Figure 4. This kind of effect is highly desired in food industry applications. Oil drop time-lapse can be observed in Figure 4 and Figure 5 for paper 1 and 2 before and after treatment in industrial plasma reactor, respectively. The oil drop did not spread much after 24 h; however, slight differences between paper 1 and 2 were observed. It seemed that oil drop on paper 1 tended to spread slower compared to the one on paper 2. This could be partially ascribed to the difference in initial microtopography of both papers, as paper 2 seemed to have deeper pores that were probably not fully covered with the HMDSO-like coating. The difference in surface properties of papers coated by two plasma reactors can also be partially ascribed to differences in surface nanotopography and density of grains and pores, however, it will become evident in the second section that surface chemistry was significantly altered due to the use of two different plasma systems at different plasma treatment conditions.

### 3.3. Surface Chemistry

#### 3.3.1. XPS Analyses

**Figure 6:**
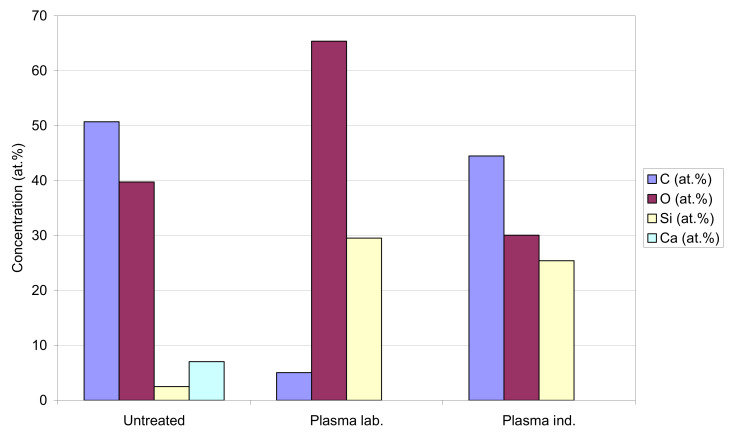
Surface composition in at.% of untreated, laboratory plasma, and industrial plasma treated samples analyzed by XPS method.

**Figure 7:**
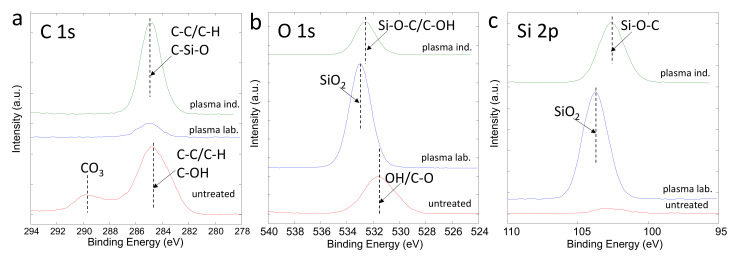
High energy resolution XPS spectra C 1s (**a**), O 1s (**b**), and Si 2p (**c**) from untreated (read line), laboratory plasma (blue line), and industrial plasma (green line) treated samples.

XPS analyses were performed to get insight into surface chemistry. Figure 6 and Table 2 show surface composition in at.%, obtained by XPS method, for paper 1. Figure 7a–c show a stack of high energy resolution XPS spectra C 1s and O 1s from untreated paper, a laboratory-plasma treated paper, and an industrial-plasma treated paper. The C 1s spectrum from untreated paper consisted of peak at 284.8 eV assigned to C–C/C–H bonds and a peak at 286.0 eV assigned to the C–OH bonds. In addition, there was a notable peak at 289.5 eV, which originated from CO_3_ bonds (CaCO_3_ particles). The O 1s spectrum of the untreated sample was at 531.5 eV, which may be assigned to OH/C–O bonds present in the coating of the untreated paper. Similar atomic concentration of C, O, and Si were detected for paper 2, while in this case a much lower amount of Ca was detected (about 2 at%). The laboratory-plasma treated sample showed XPS spectra characteristic for SiO_2_-like coating; the surface composition (Figure 6) mainly reassembled the pure SiO_2_ coating, the O 1s peak was at 533.2 eV, and Si 2p peak was at 103.5 eV, and both were assigned to SiO_2_ bonds. The SiO_2_-like coating seemed to completely cover the paper surface since no signal of Ca was detected on the plasma treated paper. The industrial-plasma treated paper had a different surface layer than the laboratory-plasma treated one, which can be recognized from surface composition in Figure 6 as well as from the different shape of the XPS spectra. The concentrations of C (45 at.%), O (30 at.%), and Si (25 at.%) indicated the presence of a HMDSO-like coating on the surface. The C 1s XPS spectrum positioned at 285.0 eV, O 1s spectrum at 532.6 eV, and Si 2p spectrum at 102.5 eV were related to the formation of the C–Si–O bonds, which confirms the HMDSO-like coating on the industrial-plasma treated paper. Similar effects after treatment in laboratory plasma and industrial plasma reactor were observed also in the case of paper 2, but results are not shown.

#### 3.3.2. SIMS Analysis

**Figure 8:**
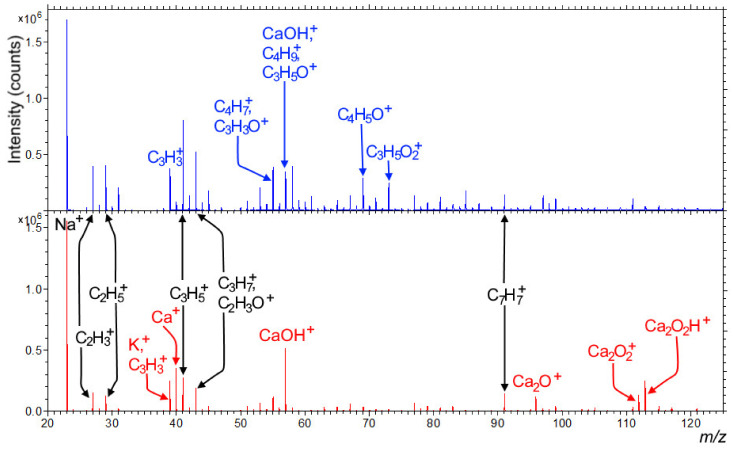
Positive secondary ion spectra of paper 2 (**upper**, blue spectrum) and paper 1 (**bottom**, red spectrum) in the *m/z* range from 20 to 125. The most important peaks were assigned with the blue color representing only paper 2, red only paper 1, and black signals equivalently found in both of the papers.

Further analysis of the coating was conducted by SIMS on both papers before and after treatment in two types of plasma reactors. Positive secondary ion spectra show the difference between different types of papers. Paper 1 was enriched with calcium salts; while on paper 2, different C, O, and H based fragments were detected, originating from the cellulose. The positive spectra of both papers are presented in Figure 8. The negative secondary ion spectra, on the other hand, showed no significant difference, with mainly cellulose fragments and some sulfonate detergents for both paper types (data not shown).

**Figure 9:**
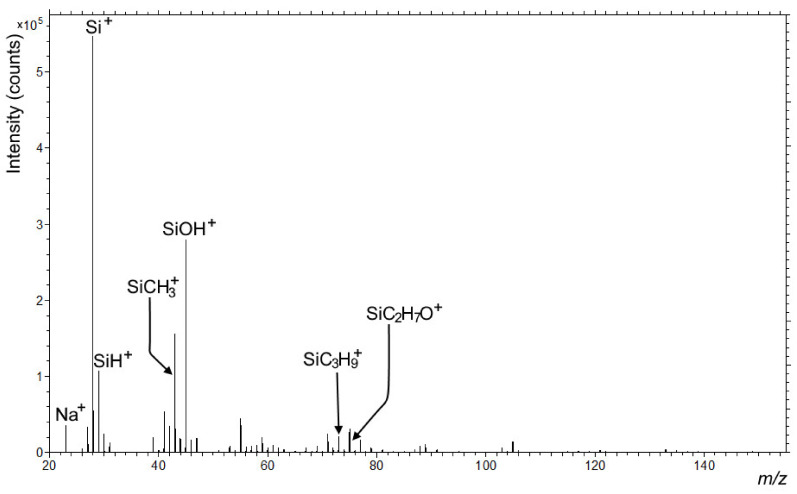
Positive secondary ion spectrum of the paper 1 coated with HMDSO in laboratory plasma reactor in the *m/z* range from 20 to 155.

SIMS analysis on paper 1 and 2 coated with HMDSO by laboratory plasma reactor show that the surface was covered by a SiO_2_ layer. HMDSO seemed to convert into a SiO_2_ layer, an intense signal for the SiOH^+^ fragment was observed, which was also confirmed by XPS analysis. In Figure 9, spectra of the positive secondary ions emitted from the surface of the laboratory plasma treated paper 1 was presented, and similar results were obtained also for paper 2 (data not shown).

**Figure 10:**
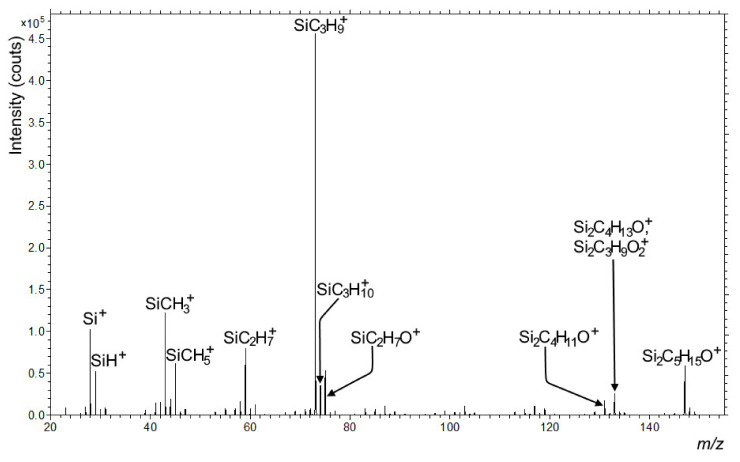
Positive secondary ion spectrum of the paper 2 coated with HMDSO in industrial plasma reactor in the *m/z* range from 20 to 155.

In contrast, papers coated with HMDSO in the industrial plasma reactor exhibit very different surface composition compared to papers treated in laboratory plasma reactor. The spectrum of positive secondary ions emitted from paper 1 coated in industrial plasma reactor is shown in Figure 10. In this case, mainly SiCH_5_^+^ fragments were detected at the same nominal mass as the SiOH^+^ fragment before. The intensity of the SiOH^+^ is very low, indicating the absence of SiO_2_. Furthermore, many positive secondary ions originating from organosilicon compounds were seen in the spectrum (Figure 10). Some organosilicon fragments we also found in the spectrum in Figure 9. However, they were less prominent compared to the ones detected in Figure 10.

**Figure 11:**
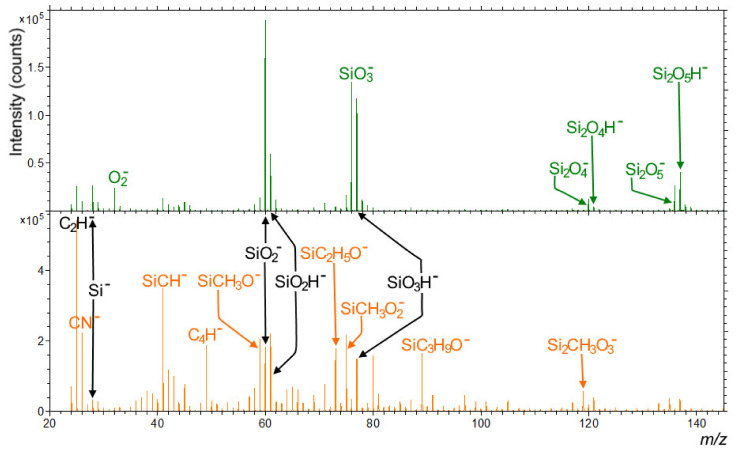
Negative secondary ion spectra of the HMDSO covered paper 1 by laboratory plasma reactor (**upper**, green spectrum) and the one treated in an industrial plasma reactor (**bottom**, orange spectrum) in the *m/z* range from 20 to 145. The most important peaks are assigned, with the green color representing only paper 1 treated in laboratory plasma reactor, orange representing only paper 1 treated in the industrial plasma reactor, and black representing signals equivalently found in both cases of the plasma treatment.

Even more pronounced differences between papers coated with HMDSO using the laboratory plasma and the industrial plasma reactor can be observed at the negative SIMS spectra. As it can be seen in Figure 11 (upper, green spectrum), only Si_x_O_y_^−^ and Si_x_O_y_H^−^ fragments, originating from the SiO_2_ layer (besides C_2_H^−^, Si^−^ and O_2_^−^), can be found when analyzing laboratory plasma treated paper. On the other hand, when the industrial plasma reactor was used (Figure 11, bottom, orange spectrum), only SiO_2_^−^, SiO_2_H^−^, and SiO_3_H^−^ fragments were present, but with much lower intensity than in the case of laboratory plasma reactor. Most of the other signals belong to the organosilicon fragments originating from the HMDSO. It must also be emphasized that comparing two different types of HMDSO coated papers was not problematic, as SIMS was a surface-sensitive technique where only a few topmost atomic/molecular monolayers with a thickness of approximately 2 nm were analyzed. Thus, no information about the underlying paper was gathered during the surface spectra analysis.

**Figure 12:**
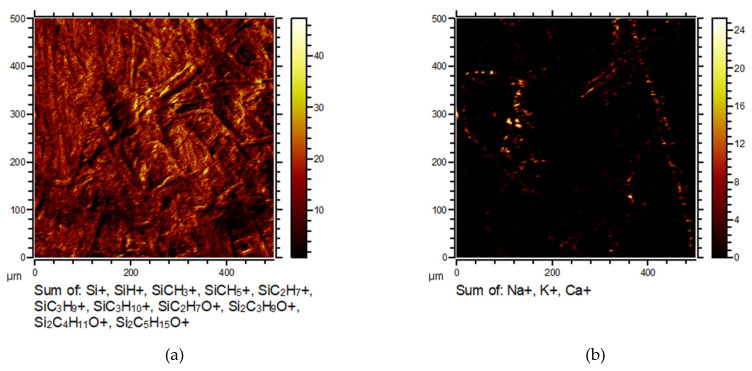
Micrographs of positive secondary ions emitted from paper 1 covered with HMDSO like layer by industrial plasma reactor. On the micrograph (**a**) are ions representing the HMDSO layer and on the micrograph (**b**), Na, K, and Ca ions.

To present the uniformity of the coating, micrographs were taken as well and are shown in Figure 12. It was evident from these micrographs that there was a more or less uniform HMDSO layer spread over the whole surface of the paper, as it can be seen in the left micrograph (paper 1 treated in industrial plasma reactor) in Figure 12. There were still some areas with increased concentration of Na, K, and Ca (right micrograph in Figure 12), but they were not significantly prominent. Coating with HMDSO in industrial plasma as well as in laboratory plasma (data not shown) seemed to provide uniform coverage for the relatively rough surface of the paper.

## 4. Conclusions

Two different types of papers and two different types of plasma reactors were used for coating paper with HMDSO. Results of our study show that regardless of the papers’ initial differences in morphology and chemical composition, both plasma treatments enabled uniform coverage of the paper surface. However, significant differences between the two plasma systems were observed. In the case of laboratory plasma, practically pure SiO_2_ coating was obtained on both types of papers, as determined from XPS and SIMS analysis. Surfaces were fully hydrophilic and no changes in oleophobic properties were observed. The oil drop was fully absorbed and spread on these papers, which could be partially explained by the fragile SiO_2_ coating formed on the surface. In the case of industrial plasma, surfaces were coated by a HMDSO-like coating, which increased its hydrophobic and oleophobic properties. The oil drop was not absorbed into the paper; even after 24 h of contact, only slight absorption on the edges was observed. Slight differences in absorption of oil were, however, observed between two different types of paper. Overall, HMDSO-like coating of papers could present an interesting approach to alter surface properties of paper for specific applications, like for the food packaging industry. Increasing coating thickness could further improve the oleophobicity as well as barrier properties. However, treatment times should be significantly reduced to reach the demand for rapid treatment conditions used in paper industry.

## Figures and Tables

**Table 1:** The results of water contact angles and surface energy measurements of paper 1 and 2 in untreated state and immediately after plasma coating treatment in laboratory and industrial reactor.

	Paper 1			Paper 2		
	WCA (°)	SE (mN/m)	CA of Oil (°)	WCA (°)	SE (mN/m)	CA of Oil (°)
Untreated	71.4 (±6.7)	48.8 (±3.9)	<5	115.0 (±4.0)	31.2 (±1.3)	<5
Laboratory plasma	21.4 (±0.9)	69.8 (±2.5)	<5	41.0 (±2.8)	57.4 (±2.3)	<5
Industrial plasma	124.9 (±2.5)	11.6 (±2.6)	59.2 (±1.8)	109.8 (±7.9)	14.4 (±2.5)	68.5 (±3.4)

**Table 2:** Surface composition in at.% obtained by XPS analyses.

Sample	C (at. %)	O (at. %)	Si (at. %)	Ca (at. %)
Untreated	50.7	39.8	2.5	7.0
Laboratory plasma	5.1	65.4	29.6	0.0
Industrial plasma	44.5	30.1	25.4	0.0
